# Novel Mesoporous Cetyltrimethylammonium Bromide-Modified Magnetic Nanomaterials for Trace Extraction and Analysis of Bisphenol Endocrine Disruptors in Diverse Liquid Matrices

**DOI:** 10.3390/molecules30030628

**Published:** 2025-01-31

**Authors:** Yichao Gong, Yajing Guo, Qizhi Sun, Pengyan Liu

**Affiliations:** 1 College of Chemical Engineering and Biotechnology, Xingtai University, Xingtai 054001, China; 2 School of Clinical Medicine, Xingtai Medical College, Xingtai 054000, China; 3 School of Eco-Environment, Hebei University, Baoding 071000, China; 18730262608@163.com (Q.S.); hbupyliu@163.com (P.L.)

**Keywords:** mesoporous materials, trace extraction, bisphenol endocrine disruptors, environmental water samples, food samples

## Abstract

In this study, Fe_3_O_4_ was used as a magnetic core, combined with the characteristics of mesoporous adsorbents, to prepare a novel magnetic mesoporous composite material named MMC. Cetyltrimethylammonium bromide (CTAB) and tetraethyl orthosilicate (TEOS) were used as functional monomers, and a simple etching method was employed. The resulting MMC was used as an effective adsorbent for the magnetic solid-phase extraction of trace residues of six bisphenol endocrine disruptors (bisphenol A, bisphenol B, bisphenol C, bisphenol F, bisphenol AF, and bisphenol AP) from environmental water and food samples. Characterization results indicated that the surface of MMC exhibited a distinct wormhole-like mesoporous structure, with the successful incorporation of CTAB functional groups and Si-OH. The crystal structure of Fe_3_O_4_ remained stable throughout the preparation process. Mapping analysis confirmed the uniform distribution of CTAB functional groups without aggregation and demonstrated high magnetic intensity, enabling rapid separation and collection under an external magnetic field. Extraction and elution conditions were optimized, and tests were conducted for interfering substances such as humic acid, glucose, fructose, and sucrose under optimal parameters. The results showed that recovery rates were not significantly affected. The quality evaluation of the method demonstrated good linearity, a broad linear range, low limits of detection and quantification, and satisfactory recovery rates. Blank and spiked analyses were conducted for seven real samples, including environmental water (rivers and lakes) and food samples (dairy, juice, and carbonated beverages), with satisfactory spiked recovery rates achieved. Thus, the developed analytical method enables the analysis and detection of trace residues of various bisphenol pollutants in complex matrices, such as environmental water and food samples, providing a valuable reference for trace analysis of similar contaminants in complex matrices.

## 1. Introduction

**Figure 1:**
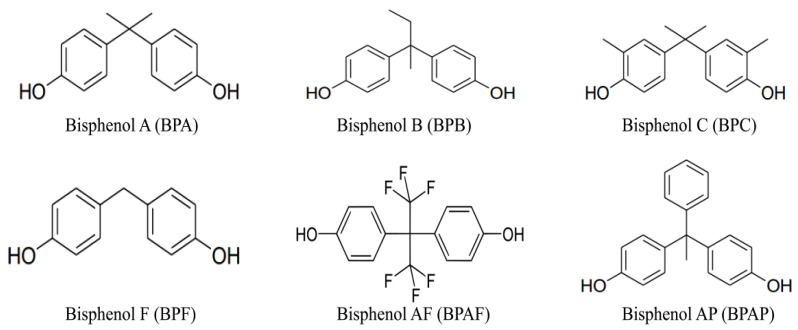
Chemical structural formulas of six bisphenol endocrine disruptors.

Endocrine-disrupting chemicals (EDCs) are exogenous substances that interfere with the human endocrine system, impeding the metabolism of steroid hormones. With the rapid advancement of industrialization, the number and variety of EDCs have been steadily increasing, exerting significant impacts on the environment, organisms, and human health [1,2]. Bisphenols (BPs) are one of the most common and abundant types of EDCs, characterized by their typical structure containing two phenolic hydroxyl groups [3]. In recent years, increasing attention has been paid to the environmental and health risks posed by bisphenol pollutants. Bisphenol A (BPA), as a representative bisphenol compound, has been shown to disrupt the healthy development of reproductive, neurological, behavioral, and cognitive functions [4,5]. Consequently, countries around the world have imposed strict regulations on the use of BPA. The U.S. Food and Drug Administration (FDA) has already banned the use of BPA in baby bottles and sippy cups [6,7]. Due to the stringent international regulations on the production and use of BPA, alternative bisphenols such as Bisphenol B (BPB), Bisphenol C (BPC), Bisphenol F (BPF), Bisphenol AF (BPAF), and Bisphenol AP (BPAP) have emerged as substitutes for BPA [8]. The chemical structures of these bisphenols are shown in Figure 1.

Due to the widespread use of Bisphenol A (BPA) and its analogs, bisphenol pollutants and their degradation products can be released into aquatic environments through atmospheric deposition, industrial wastewater discharge, runoff, urban sewage, and other pathways during production, usage, aging, disposal, and release processes [9,10]. Studies have shown the presence of bisphenol pollutants in surface waters in countries such as India, South Korea, Japan, and China [11,12]. One report found that in 25 samples collected from the Pearl River Estuary in China, Bisphenol F (BPF) was the dominant bisphenol pollutant, with an average concentration of 46.7 ng/L, followed by BPA at an average of 30.7 ng/L. It was also discovered that BPF had a higher concentration than other bisphenols in freshwater samples, and a similar trend was observed in Japan’s Tamagawa River system, where BPF reached the highest concentration of 2850 ng/L. Additionally, water samples from India showed that Bisphenol B (BPB) and Bisphenol AP (BPAP) were present at concentrations around 10 ng/L [13,14]. Due to the significant differences in the octanol-water partition coefficients of bisphenol pollutants [15], the degree of contamination varies between regions. In some areas, pollutants such as BPF, BPB, and BPAF have even exceeded BPA contamination levels [16,17]. Moreover, bisphenol contamination in food products is also a serious concern. Research has detected bisphenol pollutants in canned food, beverages, and baby bottles, primarily because epoxy resins, used as inner coatings for food cans and packaging materials, can leach bisphenols into food [18]. Recent studies have revealed the widespread usage and environmental occurrence of bisphenol analogs such as BPF and BPAP, highlighting their comparable endocrine-disrupting potential to BPA [19,20]. As regulatory agencies across regions like the EU and US increasingly restrict BPA, these emerging analogs remain largely unregulated, underscoring the urgency of expanded monitoring and risk assessment. Therefore, considering the potential harm bisphenol pollutants pose to human health, as well as their trace-level presence in both the environment and food, it is necessary to develop a simple, rapid, effective, and sensitive method for detecting trace residues of BPA, BPB, BPC, BPF, BPAF, and BPAP in environmental water samples and food products.

Due to the often-complex matrices of environmental and food samples, high technical requirements are necessary for sample separation, extraction, enrichment, and purification, making the choice of detection techniques and pretreatment methods critical for accurate analysis [7,21]. High-performance liquid chromatography (HPLC) is widely used for its broad range of target analytes and low detection limits. However, the fluorescence detector (FLD) offers advantages such as high sensitivity and low cost, with detection sensitivity that is 2–3 orders of magnitude higher than that of commonly used ultraviolet detectors. Since FLD only responds to compounds with fluorescent properties, and most bisphenols exhibit fluorescence, the combination of HPLC-FLD for bisphenol determination not only enhances sensitivity but also reduces interference from other impurities [22]. Bisphenol pollutants typically exist in trace amounts in environmental water and food samples, and due to the complexity of these matrices, direct measurement is not possible. Therefore, sample pretreatment is required before analysis. In recent years, magnetic solid-phase extraction (MSPE) has gained attention because it allows for the rapid separation of adsorbents and solutions using an external magnetic field without the need for cumbersome centrifugation during the adsorption, enrichment, and elution processes. Magnetic adsorbents are also easy to clean and can be recycled and reused, making MSPE operations simpler and more efficient. This approach effectively prevents the loss of target compounds due to incomplete separation, thereby improving recovery rates [23,24].

Mesoporous adsorbents can significantly improve adsorption capacity by increasing the number of exposed active sites and reducing mass transfer resistance, thereby shortening the adsorption equilibrium time. Silica-based mesoporous adsorbents are currently a well-researched and widely applied class of materials [25]. However, unmodified mesoporous silica adsorbents, despite having a large specific surface area, have limited adsorption capacity for organic compounds due to their simple functional groups, primarily silanol groups. Therefore, surface modification is the most effective and direct method for enhancing the adsorption performance of mesoporous adsorbents. Cetyltrimethylammonium bromide (CTAB), a highly efficient quaternary ammonium cationic surfactant, is commonly used to prepare ordered mesoporous silicate molecular sieves [26]. The surface of silica-coated Fe_3_O_4_ contains abundant Si-OH groups, which can readily bind with CTAB groups on its surface [27,28]. Additionally, Fe_3_O_4_ magnetic cores exhibit excellent superparamagnetic properties, enabling the rapid separation and collection of the adsorbent from water under a magnetic field [29], making SiO_2_-coated Fe_3_O_4_ an ideal carrier. Moreover, due to the presence of hydroxyl groups and benzene rings in the structure of bisphenol pollutants and the electrostatic and hydrophobic properties of CTAB groups [30,31], these interactions facilitate the formation of hydrogen bonds, electrostatic interactions, and hydrophobic interactions with bisphenols. Because CTAB is an ionic surfactant, using it as a template in mesoporous material synthesis usually leads to its removal during the template extraction step, thereby greatly reducing the material’s adsorption capacity. By contrast, a non-template approach that employs selective etching proves both simpler and more efficient in retaining functional sites within the mesoporous framework. In recent years, the successful preparation of hollow mesoporous silica microspheres using the etching method has been reported [32,33], making it feasible to use etching to prepare CTAB-functionalized magnetic mesoporous materials.

In this study, a novel magnetic composite material, termed MMC, was synthesized using a combination of sol–gel and simple etching techniques. The material demonstrated excellent performance in the trace extraction and analysis of various bisphenol pollutants (BPA, BPB, BPC, BPF, BPAF, and BPAP) in complex matrices. By systematically optimizing the extraction and elution conditions, a highly sensitive MSPE–HPLC-FLD method was established and successfully applied to the analysis of water and food samples. The simple preparation process and high separation efficiency of MMC offer new possibilities for environmental and food safety monitoring.

## 2. Results and Discussion

### 2.1. Preparation and Characterization

**Figure 2:**
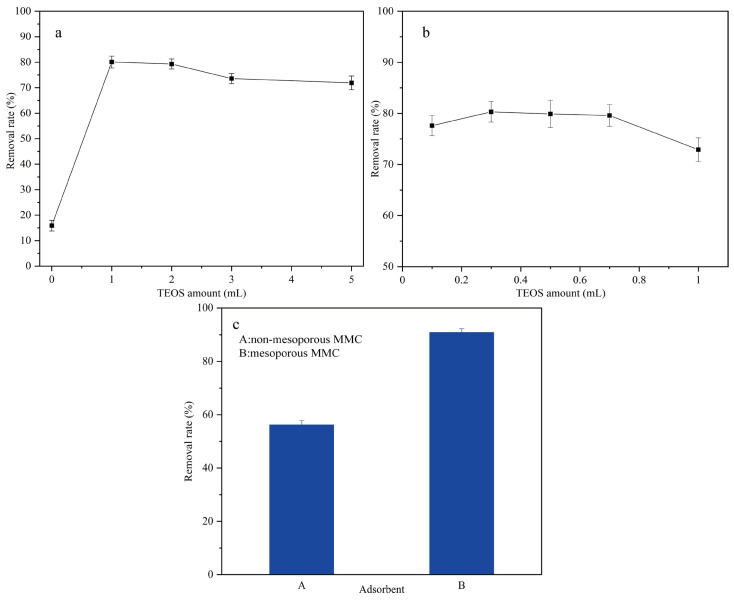
Effect of TEOS amount on the BPA removal by CTAB-functionalized mesoporous and non-mesoporous adsorbents ((**a**) TEOS amount in the process of preparing silica-coated Fe_3_O_4_; (**b**) TEOS amount in the process of preparing MMC; (**c**) removal rate of mesoporous and non-mesoporous materials).

The adsorption performance of MMC primarily relies on the interaction between Si-OH groups and CTAB for functionalization, as well as the pore formation through alkaline etching, which creates a mesoporous structure. Therefore, the amount of TEOS added during the preparation of silica-coated Fe_3_O_4_ is critical, as it directly influences the number of Si-OH groups. The second addition of TEOS is aimed at attaching CTAB functional groups in preparation for the subsequent pore formation via weak alkaline etching. The use of Na_2_CO_3_ as the etching agent and adjusting the pH to 10–11 is essential because, in a weak alkaline environment, it allows the construction of mesoporous structures on the adsorbent surface without damaging the SiO_2_ shell framework. This study focused on investigating the amount of TEOS added twice, while keeping the CTAB amount constant (400 mg). The effect of different amounts of TEOS on the adsorption capacity was studied under the same adsorption conditions, and the results are shown in Figure 2. As shown in Figure 2a, during the preparation of silica-coated Fe_3_O_4_, the adsorption capacity for BPA initially increases and then stabilizes with the increasing amount of TEOS. This is because when no TEOS is added, the functionalization process primarily involves the interaction between Fe-OH groups and CTAB. However, the relatively low number of Fe-OH groups, combined with the instability of the Fe-OH-CTAB complex, results in decreased adsorption capacity. On the other hand, excessive TEOS addition leads to an excess of Si-OH groups on the surface of silica-coated Fe_3_O_4_, which can cause the aggregation of CTAB functional groups, reducing their ability to effectively adsorb BPA, and consequently, affecting the adsorption performance. The second addition of TEOS is intended to form mesoporous structures. As shown in Figure 2b, a small amount of TEOS enhances the adsorption performance, while excessive TEOS may lead to incomplete weak alkaline etching, preventing the full exposure of the CTAB functional groups and thus compromising the adsorption effectiveness. Therefore, in the subsequent preparation of silica-coated Fe_3_O_4_ and MMC, the amounts of TEOS added were 1 mL and 0.3 mL, respectively. To confirm that the mesoporous structure of MMC enhances its adsorption performance, the adsorption capacities of CTAB-functionalized mesoporous and non-mesoporous adsorbents for BPA were compared. The results are shown in Figure 2c. As observed, the CTAB-functionalized mesoporous composite significantly increased the BPA removal rate by approximately 30%. This indicates that the mesoporous structure substantially improves the adsorption performance. Details regarding the adsorption types can be found in the Supplementary Materials.

The morphology of Fe_3_O_4_, silica-coated Fe_3_O_4_, and MMC was determined using SEM and TEM, and the results are shown in Figures S3 and S4. Figures S3a and S4a demonstrate that Fe_3_O_4_ exhibits a monodispersed spherical structure with a diameter of approximately 100–200 nm, and its surface is relatively rough. Figures S3b and S4b show that after SiO_2_ is coated on the surface of Fe_3_O_4_, silica-coated Fe_3_O_4_ increase in size, and their surface becomes smoother. The diameter increases by about 10–20 nm, and uncoated SiO_2_ is also observed around silica-coated Fe_3_O_4_. The thickness of the SiO_2_ surface layer on silica-coated Fe_3_O_4_ is approximately 5–10 nm. Figures S3c and S4c demonstrate that due to the secondary SiO_2_ coating, the diameter of MMC continues to increase, and after treatment with a weak alkaline etching method, the surface exhibits a distinct wormhole-like mesoporous structure. The mesoporous structure offers advantages such as reduced mass transfer resistance and an increased number of active sites, thereby enhancing the adsorption capacity of the adsorbent and shortening the adsorption equilibrium time.

A mapping analysis of the main elements on the surface of MMC was conducted using spherical aberration-corrected field emission electron microscopy, and the mapping results are presented in Figure S5a. From the figure, it can be concluded that the aggregation of iron (Fe) is due to the magnetic core of Fe_3_O_4_ in MMC. Silicon (Si) and carbon (C) elements were observed both on Fe_3_O_4_ and around its surface, which is attributed to the addition of TEOS and CTAB during MMC preparation, both of which contain carbon elements. Bromine (Br), a characteristic element of CTAB, was evenly distributed in MMC, indicating that the CTAB functional groups were well dispersed without aggregation. To further verify the elemental composition and content of MMC and confirm successful preparation, energy-dispersive X-ray spectroscopy (EDS) analysis was conducted, with the results shown in Figure S5b. The EDS spectrum in Figure S5b reveals that MMC contains C, N, O, Si, Br, and Fe elements, with Fe having the highest content at approximately 65.73%, and Br the lowest at around 1.78%. The C, N, O, and Si contents were approximately 3.53%, 2.05%, 22.94%, and 3.96%, respectively. Since Si and Br are specific elements from TEOS and CTAB, and the presence of Fe in MMC confirms that its magnetic properties were retained during subsequent modification processes. The successful synthesis of the MMC composite material was further validated.

**Figure 3:**
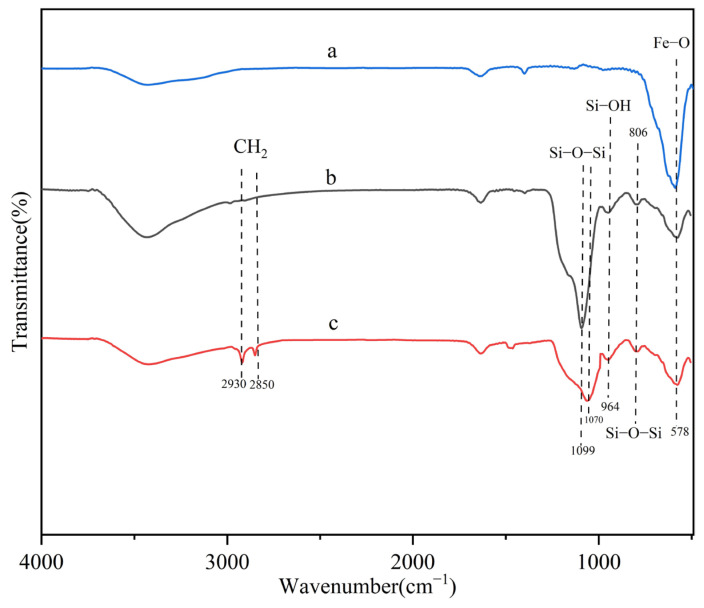
FTIR spectra of Fe_3_O_4_ (a), silica-coated Fe_3_O_4_ (b), and MMC (c).

The FTIR characterization of Fe_3_O_4_, silica-coated Fe_3_O_4_, and MMC was conducted, and the results are shown in Figure 3. Panels a, b, and c represent the FTIR spectra of Fe_3_O_4_, silica-coated Fe_3_O_4_, and MMC, respectively. As shown in the figure, the peak at 578 cm^−1^ corresponds to the stretching vibration of Fe-O-Fe [34], confirming the presence of Fe_3_O_4_ in both Fe_3_O_4_ and MMC. In Figure 3b, the peaks at 1099 cm^−1^ and 964 cm^−1^ are attributed to the asymmetric stretching vibration of Si-O-Si and the stretching vibration of Si-OH [35], indicating the successful incorporation of TEOS onto the surface of Fe_3_O_4_. For pure CTAB, typical peaks are observed around 2917 cm^−1^ and 2850 cm^−1^, corresponding to the asymmetric and symmetric stretching vibrations of CH_2_ [36]. In Figure 3c, the peaks at 2850 cm^−1^ and 2930 cm^−1^ are caused by the asymmetric stretching vibrations of CH_2_, indicating that the original CTAB structure remains intact in the prepared MMC. However, due to the electrostatic interactions between CTAB and Si-OH groups, the Si-O-Si peak of MMC shifts to 806 cm^−1^ and 1070 cm^−1^ compared to silica-coated Fe_3_O_4_.

**Figure 4:**
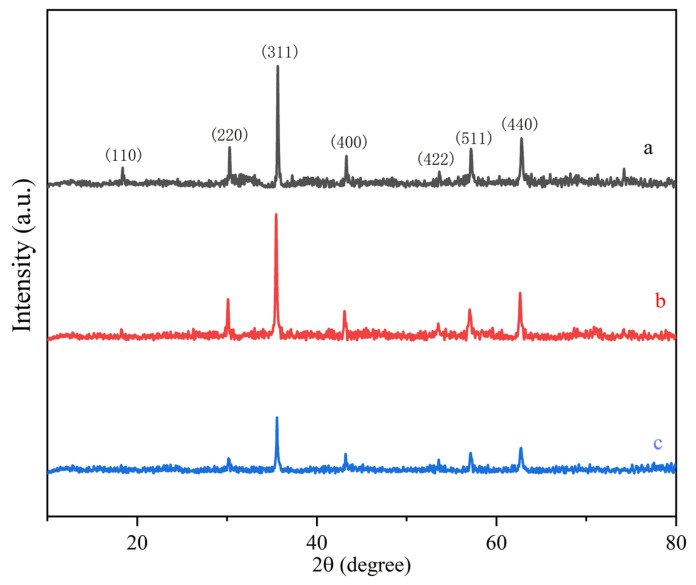
XRD patterns of Fe_3_O_4_ (a), silica-coated Fe_3_O_4_ (b), and MMC (c).

XRD was used to analyze the crystal structure of Fe_3_O_4_, silica-coated Fe_3_O_4_, and MMC, and the results are shown in Figure 4. Figure 4a shows that Fe_3_O_4_ exhibits eight distinct diffraction peaks at 18.3° (110), 30.1° (220), 35.5° (311), 43.2° (400), 53.6° (422), 57.1° (511), and 62.7° (440), which are in good agreement with the standard diffraction pattern of Fe3O4 (ICDD/JCPDS card, file no. 88-0315). This confirms that Fe_3_O_4_ is pure and exhibits a cubic crystal structure. From Figure 4b,c, it can be observed that the crystal structure of silica-coated Fe_3_O_4_ and MMC retains the Fe_3_O_4_ core; however, the peak intensities are significantly reduced due to the coating of SiO_2_ and the introduction of CTAB. This confirms that we successfully synthesized magnetic materials with an Fe_3_O_4_ core in this study.

**Figure 5:**
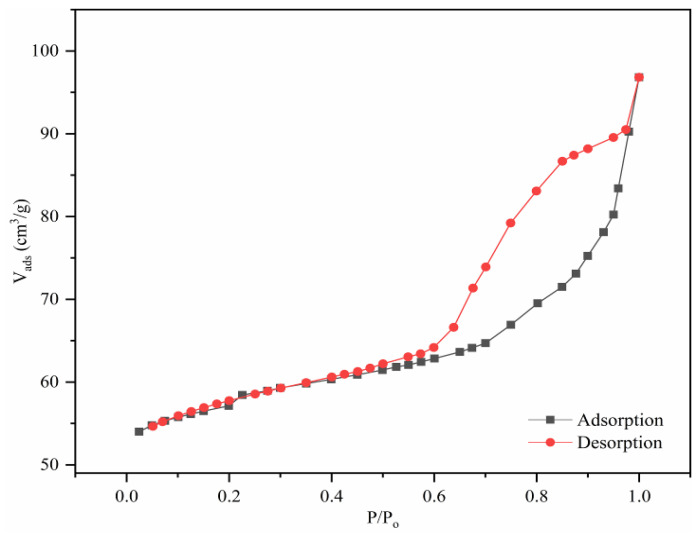
Nitrogen adsorption–desorption isotherm of MMC.

The nitrogen adsorption–desorption capacity of MMC was tested using a fully automated gas adsorption analyzer to study its characteristics such as average pore size and specific surface area. The nitrogen adsorption–desorption isotherms are shown in Figure 5. The analysis results indicate that the composite material exhibits type IV adsorption–desorption isotherms, confirming that MMC has a mesoporous structure. According to BET analysis, the average pore size of MMC is 2.9 nm and the average specific surface area is 148.6 m^2^/g. Therefore, the sol–gel method combined with weak alkaline etching successfully produced a CTAB-functionalized composite material with a mesoporous structure.

**Figure 6:**
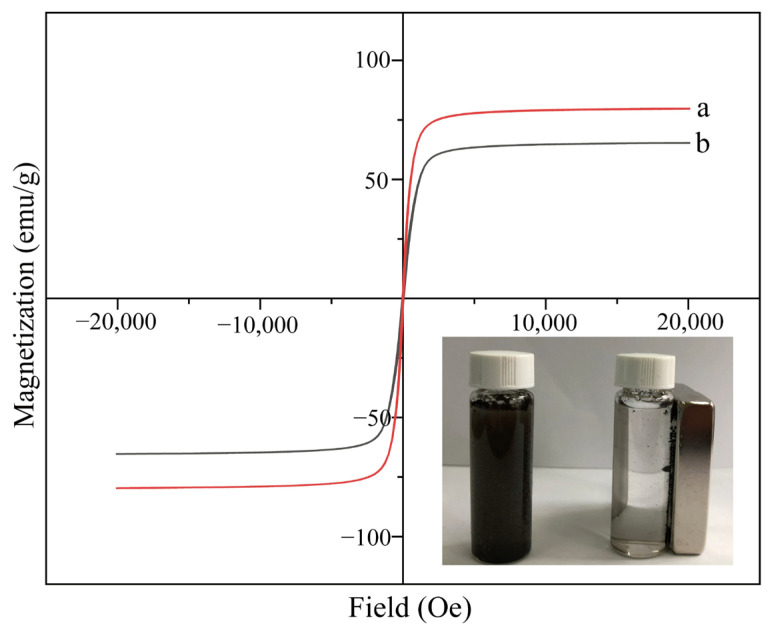
Magnetization versus magnetic field dependence of Fe_3_O_4_ (a) and MMC (b).

Figure 6 shows the magnetization versus magnetic field dependence of Fe_3_O_4_ and MMC at room temperature. As observed in Figure 6, the curves exhibit a reversible ‘S’ shape, with both coercivity and remanent magnetization of Fe_3_O_4_ and MMC being close to zero. This indicates that both Fe_3_O_4_ and MMC retain their superparamagnetic properties before and after modification. The saturation magnetization of MMC is approximately 65.3 emu/g, which is lower than the 79.6 emu/g of Fe_3_O_4_. This reduction is attributed to the coating of Fe_3_O_4_ with SiO_2_ and the introduction of CTAB. However, MMC still exhibits relatively high saturation magnetization. Due to its superparamagnetic properties, high saturation magnetization, and rapid response to external magnetic fields, MMC can be quickly separated from solution using an external magnet.

### 2.2. Optimization of MSPE

#### 2.2.1. Optimization of Amount of CTAB Added

**Figure 7:**
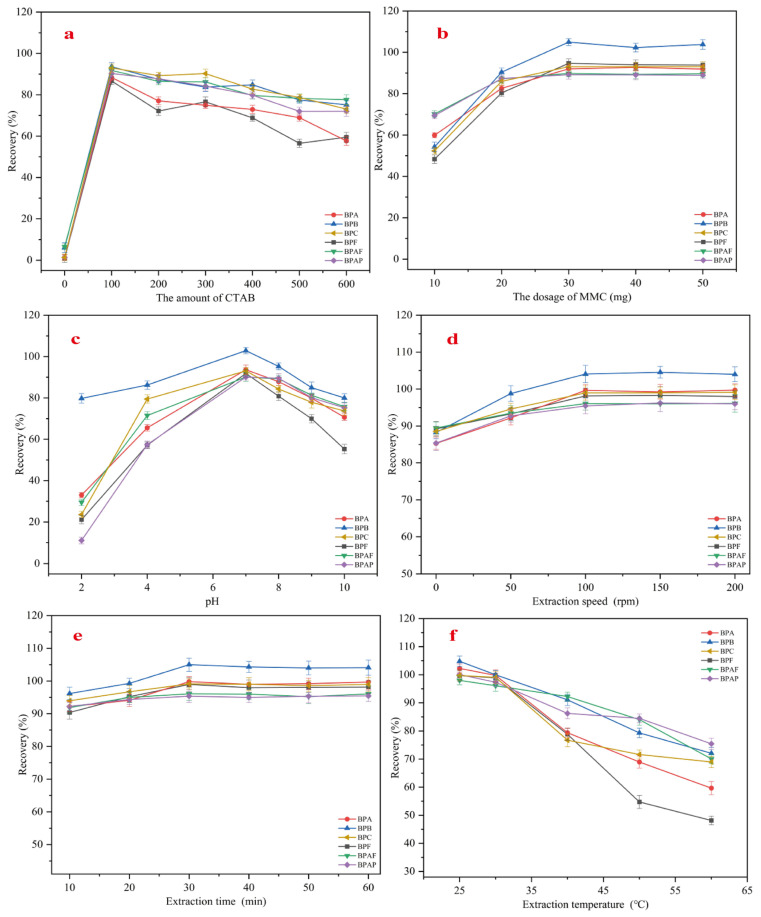
Optimization of amount of CTAB added (**a**); optimization of MMC dosage (**b**); optimization of sample pH (**c**); optimization of extraction speed (**d**); optimization of extraction time (**e**); optimization of extraction temperature (**f**).

The primary adsorption mechanism in MMC involves electrostatic and hydrophobic interactions between CTAB and bisphenol pollutants. Considering that different amounts of CTAB added to MMC may affect the extraction efficiency of bisphenol pollutants, an investigation was conducted to study the effect of CTAB amounts ranging from 0 to 600 mg on the recovery rates of six bisphenol pollutants, as shown in Figure 7a. The results indicate that the amount of CTAB has a significant impact on extraction efficiency. When the CTAB addition was 100 mg, the recovery rates of all six bisphenol pollutants were at their highest. However, as the amount of CTAB increased, the recovery rates showed a declining trend. This may be due to excessive CTAB blocking the mesoporous structure, reducing the number of available adsorption sites, and thus affecting the trace extraction recovery rates. Additionally, with higher amounts of CTAB, the CTAB functional groups in MMC may aggregate due to electrostatic interactions, further impacting trace extraction. Therefore, for subsequent pretreatment experiments, 100 mg of CTAB was used.

#### 2.2.2. Optimization of MMC Dosage

The amount of adsorbent plays a critical role in the MSPE process. To achieve optimal recovery rates and maximize the extraction efficiency of MMC, the effect of MMC dosage in the range of 10–50 mg on the recovery rates of six bisphenol pollutants was investigated, and the results are shown in Figure 7b. As the amount of MMC increased, the recovery rates of all six bisphenol pollutants also increased. When the amount of MMC reached 30 mg, the recovery rates of BPA, BPB, BPC, BPF, BPAF, and BPAP reached their maximum, with values of 94.7%, 104.9%, 93.1%, 92.0%, 89.8%, and 89.2%, respectively. However, further increases in MMC dosage led to a plateau in recovery rates. Therefore, 30 mg was chosen as the optimal dosage for extraction.

#### 2.2.3. Optimization of Sample pH

The effect of pH on the recovery rates was investigated within the pH range of 2–10. A 0.1 M NaOH and HCl solution was used as the acid–base regulator to adjust the pH, which was measured using a pH meter. The results of the recovery rate variations are shown in Figure 7c. When the pH was 7, the recovery rates of the six bisphenol pollutants were at their highest. As the pH increased from 2 to 7, the recovery rates gradually increased, but as the pH increased from 7 to 10, the recovery rates gradually decreased. Since all six bisphenol pollutants contain hydroxyl groups, they can become protonated under highly acidic conditions. In such conditions, the surface of MMC is positively charged, causing electrostatic repulsion between MMC and the bisphenol pollutants, leading to lower recovery rates. However, when the pH of the solution exceeds 7, the bisphenol pollutants become ionized, and the negatively charged MMC surface repels the ionized pollutants, resulting in a gradual decrease in recovery rates as the pH increases. Therefore, in subsequent experiments, the solution pH was adjusted to 7.

#### 2.2.4. Optimization of Extraction Speed, Extraction Time, and Extraction Temperature

To study the effect of extraction stirring speed on recovery rates, the impact of stirring speeds ranging from 0 to 200 rpm on the recovery rates of six bisphenol pollutants was investigated, and the results are shown in Figure 7d. As the stirring speed increased, the recovery rates gradually rose. When the stirring speed reached 100 rpm, the maximum extraction recovery rates for all six bisphenol pollutants exceeded 95%. Further increasing the stirring speed did not significantly affect the recovery rates, which tended to stabilize. Therefore, 100 rpm was determined to be the optimal extraction speed. To reduce extraction time and improve efficiency, the effect of extraction time (ranging from 10 to 60 min) on the recovery rates of the six bisphenol pollutants was examined, and the results are shown in Figure 7e. At 10 min, the recovery rates for all six bisphenol pollutants were relatively high, all exceeding 90%. When the extraction time reached 30 min, the recovery rates were maximized. Further extending the extraction time did not result in significant changes, as the recovery rates stabilized. Therefore, 30 min was selected as the optimal extraction time for MMC. The effect of extraction temperature (25–60 °C) on the recovery rates of the six bisphenol pollutants was also studied, and the results are shown in Figure 7f. As the temperature increased, the recovery rates tended to decrease, with BPF showing the greatest decline and BPAP the least. At 25 °C, the recovery rates for all bisphenol pollutants were at their highest, which is consistent with the fact that the adsorption process of MMC is exothermic. Therefore, subsequent extraction experiments were conducted at 25 °C to achieve optimal results.

#### 2.2.5. Optimization of Ionic Type and Strength, Eluent, and Eluent Dosage

**Figure 8:**
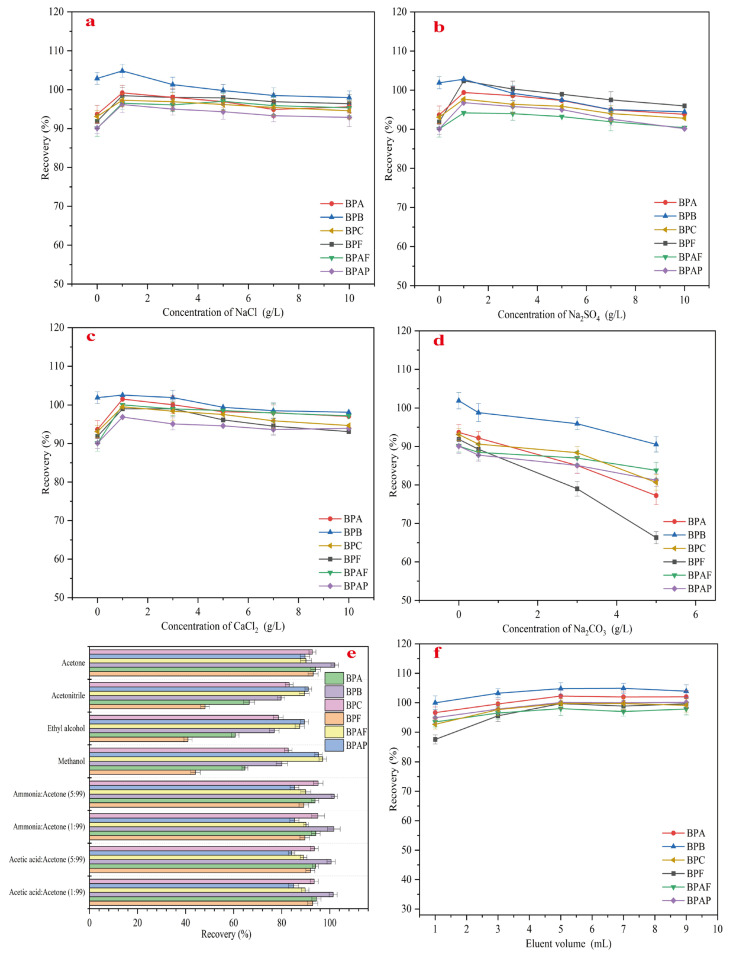
Effect of ionic type and strength (**a**) NaCl; (**b**) Na_2_SO_4_; (**c**) CaCl_2_; (**d**) Na_2_CO_3_), eluent (**e**), and eluent dosage (**f**).

The type and strength of ions can affect the hydrophobicity of the target compounds as well as the active sites on the surface of the adsorbent. To investigate this, the effect of four inorganic salts (ranging from 0 to 10 g/L) on the recovery rates of six bisphenol pollutants was studied, with the results shown in Figure 8a–d. As shown in the figures, the addition of small amounts of the three inorganic salts (except for Na_2_CO_3_) increased the recovery rates of the target compounds. The maximum recovery rates of NaCl, Na_2_CO_3_, and CaCl_2_ for the six bisphenol pollutants ranged from 96.2% to 104.8%, 94.2% to 102.4%, and 96.9% to 102.6%, respectively. A comparison of these maximum recovery rates reveals that adding Na_2_CO_3_ significantly increased the recovery of BPF but had minimal effects on the other five bisphenols. On the other hand, adding CaCl_2_ led to a significant increase in the recovery of BPAF and BPAP, but had a lesser effect on BPB. This may be due to Ca^2+^ and SO_4_^2−^ altering the surface charge of the adsorbent, thus affecting the recovery rates. However, the addition of CO_3_^2−^ can cause the ionization of bisphenol pollutants, resulting in electrostatic repulsion with MMC, which negatively impacts the extraction recovery rates of the six bisphenol endocrine-disrupting chemicals (EDCs) when Na_2_CO_3_ is added. To ensure the highest recovery rates for all six bisphenol pollutants, 1 g/L of NaCl was selected as the optimal amount of inorganic salt for extraction.

The effects of acetone, acetonitrile, ethanol, and methanol as eluents on the recovery rates of the six bisphenol pollutants were also investigated, and the results are shown in Figure 8e. The results indicate that methanol, acetonitrile, and ethanol as eluents performed well only for the elution of BPAF and BPAP, while their effectiveness for the other bisphenols was limited. Ethanol had the poorest elution performance for BPF, whereas acetone proved to be the most effective eluent for all six bisphenol pollutants. Considering that the addition of acids or bases to the eluent might enhance recovery rates, acetic acid and ammonia were chosen as the acid and base agents. Specific amounts of these agents were added to acetone to prepare 1:99 ammonia–acetone solutions, 5:95 ammonia–acetone solutions, 1:99 acetic acid–acetone solutions, and 5:95 acetic acid–acetone solutions, which were then used to extract the six bisphenol pollutants. As shown in Figure 8e, the changes in recovery rates with these solutions were minimal compared to those using acetone alone. The 1:99 ammonia–acetone solution (*v*/*v*) and 1:99 acetic acid–acetone solution (*v*/*v*) showed slightly better elution performance for BPA and BPC than acetone alone, but the differences were not significant. To maximize the recovery rates for all six bisphenol pollutants and ensure simplicity in the method, acetone was chosen as the eluent for subsequent experiments.

To minimize the amount of eluent required, the eluent volume was optimized within the range of 1–9 mL, and the results are shown in Figure 8f. As the eluent volume increased, the recovery rates for all six bisphenol pollutants gradually improved, reaching their maximum at 5 mL. Further increases in the eluent volume resulted in slight decreases in the recovery rates for BPB and BPC. Therefore, to achieve the maximum recovery rates for all six bisphenol pollutants, 5 mL was selected as the optimal eluent volume.

#### 2.2.6. Optimization of Elution Speed, Elution Time, Elution Temperature and Sample Volume

**Figure 9:**
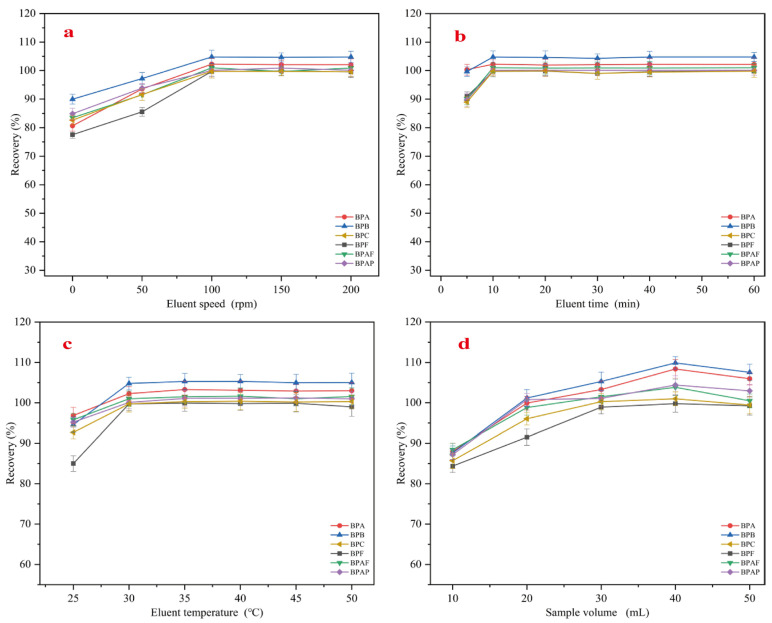
Optimization of elution speed (**a**), elution time (**b**), elution temperature (**c**), and sample volume (**d**).

The effect of elution stirring speed (ranging from 0 to 200 rpm) on the recovery rates of six bisphenol pollutants was investigated, and the results are shown in Figure 9a. The figure indicates that even under static conditions, the recovery rates of the six bisphenol pollutants were relatively high. As the stirring speed increased, the recovery rates correspondingly improved, reaching their maximum at 100 rpm. Therefore, to ensure the maximum recovery rates for all six bisphenol pollutants, 100 rpm was chosen as the optimal elution stirring speed. To save elution time and improve the efficiency of the pretreatment method, the effect of elution time (ranging from 5 to 60 min) on the recovery rates was studied, and the results are shown in Figure 9b. The figure shows that MMC achieved maximum elution of all six bisphenol pollutants within 10 min. Further increasing the elution time did not result in significant changes in recovery rates. Therefore, 10 min was selected as the shortest elution time for the six bisphenol pollutants. Since the adsorption process of MMC is exothermic, increasing the elution temperature may enhance recovery rates. Therefore, the effect of elution temperature (ranging from 25 to 50 °C) on the recovery rates was examined, and the results are shown in Figure 9c. At 30 °C, the recovery rates of five bisphenol pollutants (excluding BPAP) reached their maximum. Further increasing the temperature led to stabilization of the recovery rates, while BPAP achieved its maximum recovery at 35 °C. To ensure the maximum recovery of all bisphenol pollutants, 35 °C was selected as the optimal elution temperature.

Under the same pretreatment conditions, different sample solution volumes can have varying effects on extraction efficiency. To achieve the highest recovery rates, the effect of sample solution volume (ranging from 10 to 50 mL) on the recovery rates of the six bisphenol pollutants was investigated, with the results shown in Figure 9d. The figure indicates that the recovery rates increased initially with the sample volume but then decreased. When the sample volume reached 40 mL, the recovery rates for all six bisphenol pollutants were maximized. However, further increasing the volume to 50 mL resulted in a decline in recovery rates. Therefore, 40 mL was chosen as the optimal sample volume for trace analysis of the target compounds. The experimental results also demonstrated that MMC can achieve satisfactory recovery rates with a small sample volume, indicating the practical applicability of this adsorbent.

### 2.3. Interference Test

Natural organic matter such as humic acid is commonly present in environmental water bodies, while substances like glucose, fructose, and sucrose are often found in food samples. These components may interfere with the extraction of target compounds from real samples, potentially leading to inaccurate trace measurements or even failure in detection. Therefore, this study investigated the effect of humic acid and sugars on the extraction performance of MMC.

**Figure 10:**
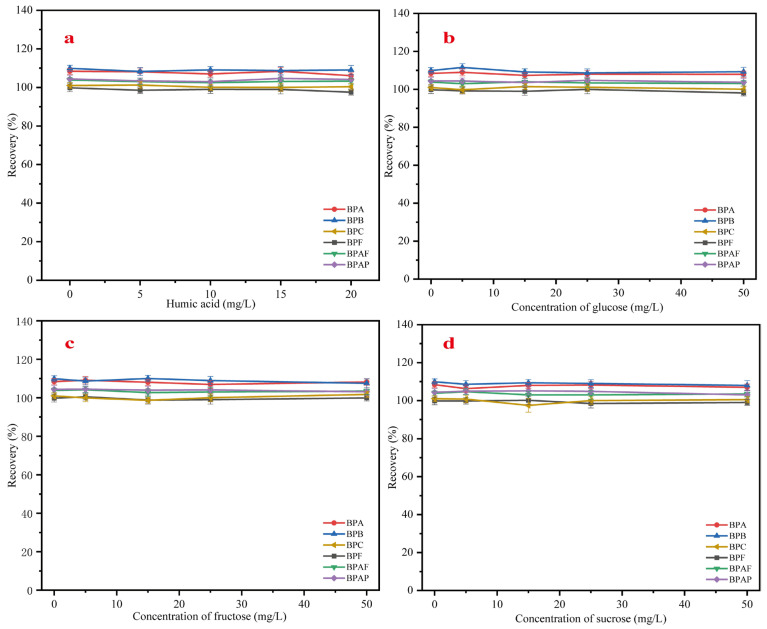
Influence of interference factors on recovery ((**a**) humic acid, (**b**) glucose, (**c**) fructose, and (**d**) sucrose).

A precise amount of sodium humate, glucose, fructose, and sucrose was weighed and prepared into 250 mL working solutions with a concentration of 10 μg/L. The concentration of sodium humate ranged from 0 to 20 mg/L, and the concentrations of the sugars ranged from 0 to 50 mg/L. Under the optimized conditions, extraction experiments were performed for each of these substances. Each interference factor was tested in triplicate, with each sample being analyzed three times. The results are shown in Figure 10. Figure 10a shows the effect of different concentrations of sodium humate on the recovery rates of the six bisphenol pollutants. It can be observed that in the range of 0–20 mg/L, humic acid had almost no effect on the recovery rates of the target compounds, indicating that MMC can be reliably used for trace analysis in real environmental samples. Figure 10b–d show the recovery rate variations in the six bisphenol pollutants in the presence of different concentrations of glucose, fructose, and sucrose, respectively. Similarly, within the concentration range of 0–50 mg/L, these sugars had a negligible effect on the extraction of the target compounds, demonstrating that MMC is also well-suited for trace extraction in real liquid food samples.

### 2.4. Method Validation

Under the optimal extraction conditions, the method’s linear range, detection limit, quantification limit, and precision were evaluated. A precise amount of standard stock solutions of the six bisphenol pollutants was measured and diluted with ultrapure water to prepare working standard solutions with concentrations ranging from 0.05 to 100 μg/L. Calibration curves were created for each of the six bisphenol pollutants, with three parallel analyses conducted for each concentration, and each analysis was repeated three times. The intra-day precision (RSD, *n* = 6) was evaluated by measuring the 10 μg/L working standard solution six times in one day, and the inter-day precision (RSD, *n* = 3) was assessed by measuring the 10 μg/L working standard solution over three consecutive days. All parameter results are shown in Table 1. As seen from the table, BPA, BPB, BPC, BPF, BPAF, and BPAP exhibited good linear relationships within the concentration range of 0.05–100 μg/L, with correlation coefficients (R) all greater than 0.997. The limits of detection (LOD) and limits of quantification (LOQ) were calculated based on signal-to-noise ratios (S/N) of 3 and 10, respectively. The LOD and LOQ values were 0.01 μg/L and 0.03 μg/L for BPA, 0.01 μg/L and 0.03 μg/L for BPB, 0.01 μg/L and 0.04 μg/L for BPC, 0.02 μg/L and 0.05 μg/L for BPF, 0.01 μg/L and 0.04 μg/L for BPAF, and 0.01 μg/L and 0.05 μg/L for BPAP. Both intra-day and inter-day precisions were less than 10.0%.

The MSPE (MMC)-HPLC-FLD method was used to continuously measure a 40 mL solution containing 10 μg/L of a mixed solution of six bisphenol pollutants six times, and the enrichment factor (*EF*) was calculated using the following mathematical expression:

$$EF=\frac{C_{a}}{C_{0}}$$

where *C_a_* is the concentration of the target compound in the final organic phase after preconcentration, which is calculated from the standard curve, and *C_0_* is the initial concentration of the target compound in the sample before pretreatment. Under optimal conditions, the calculated *EF* values ranged from 79.76 to 87.84.

### 2.5. Analysis of Real Samples

To assess the practicality and accuracy of the MSPE (MMC)-HPLC-FLD method, four environmental water samples and three food samples were analyzed. The lake water samples were collected from Baiyangdian Lake in Xiong’an New Area (Table S3), and the river water samples were taken from the Fu River in Baoding City, Hebei Province (Table S3). The milk, mango juice, and lemon-flavored soda were all purchased from a supermarket in Baoding, Hebei Province. The samples were processed according to the optimized method mentioned above, and spiked analyses at different concentrations (5 μg/L and 10 μg/L) were performed on all samples under the optimal parameters of the MSPE (MMC)-HPLC-FLD method, with three replicates for each sample. The results are presented in Table S4, and the chromatograms of the spiked samples are shown in Figure S6. As seen in Figure S6, other sample matrices caused minimal interference with the target compounds. Table S4 shows that the spiked recovery rates for real samples ranged from 68.3% to 109.8%, which is satisfactory. Clearly, the MSPE (MMC)-HPLC-FLD method established in this study demonstrates sufficient accuracy, applicability, and sensitivity for the determination of six trace bisphenol pollutants in environmental and food samples.

### 2.6. Comparison of Methods

The MSPE (MMC)-HPLC-FLD method established in this study was compared with other reported methods for the simultaneous detection of bisphenol pollutants, and the results are shown in Table 2. As can be seen from the table, compared to other analytical methods reported in the literature, the MSPE (MMC)-HPLC-FLD method offers lower detection limits and higher recovery rates. Although its detection limit is not as low as that of the MSPE-HPLC-MS method, it is still relatively close. Moreover, the preparation of MMC is straightforward and cost-effective. For trace-level bisphenol pollutants in complex matrices, the method achieves low detection limits and high sensitivity using HPLC-FLD, eliminating the need for expensive HPLC-MS instrumentation. Therefore, this method features lower detection limits, considerable recovery rates, and eliminates the need for complex and time-consuming centrifugation. It provides a new approach for the analysis of trace levels of multiple bisphenol pollutants in environmental water and food samples.

## 3. Materials and Methods

### 3.1. Reagents and Apparatus

BPA (>99%), BPB (>99%), BPC (>99%), BPF (>99%), BPAF (>99%), and BPAP (>99%) were all purchased from Aladdin Chemistry Co., Ltd. (Shanghai, China). The mixed standard stock solutions (100 mg/L) of BPA, BPB, BPC, BPF, BPAF, and BPAP were stored in dark conditions at 5 °C. Ferric chloride hexahydrate (FeCl_3_·6H_2_O, 99%), ferrous sulfate heptahydrate (FeSO_4_·7H_2_O, 99%), ammonium solution (NH_3_·H_2_O, 25%), sodium chloride (NaCl, 99%), sodium hydroxide (NaOH, 98%), hydrochloric acid (HCl, 36%), sodium carbonate (Na_2_CO_3_, 99%), calcium chloride (CaCl_2_, 99%), sodium sulfate (Na_2_SO_4_, 99%), and potassium chloride (KCl, 99%) were purchased from Damao Chemical Reagent Factory (Tianjin, China). The sodium chloride, sodium carbonate, and calcium chloride were vacuum-dried at 50 °C for 24 h before use. Tetraethyl orthosilicate (TEOS, 98%) and Cetyltrimethylammonium bromide (CTAB) were purchased from Macklin Biochemical Technology Co., Ltd. (Shanghai, China). Formic acid (HPLC grade), methanol (HPLC grade), ethanol (HPLC grade), acetone (HPLC grade), acetonitrile (HPLC grade), acetic acid (HPLC grade), humic acid (HA, 90%), glucose (98%), fructose (98%), and sucrose (98%) were purchased from Kemiou Chemical Reagent Co., Ltd. (Tianjin, China). Ultrapure water (prepared by PURELAB Classic, ELGA, High Wycombe, UK) was used in all experiments.

The morphology of the composite material was investigated using scanning electron microscopy (SEM) (S4800, Hitachi, Japan). The morphology of the composite material was investigated by transmission electron microscopy (TEM) (TECNAI G2 20, FEI, Hillsboro, OR, USA). The distribution of elements on the MMC surface was measured using spherical aberration field emission transmission electron microscopy (STEM-mapping) (Themis Z, FEI, Hillsboro, OR, USA). Energy dispersive spectroscopy (EDS) (ESCALAB 250XI, Thermo Electron, Waltham, MA, USA) was used to analyze the element types and contents of MMC. Fourier transform infrared spectroscopy (FTIR) (NICOLET 5700, Thermo Electron, Waltham, MA, USA) was utilized to identify the surface functional groups using KBr pelleted samples. X-ray diffraction (XRD) patterns were obtained using an XRD analyzer (D8 ADVANCE, Bruker, Germany) utilizing Cu radiation at 40 kV and 40 mA. Brunauer–Emmett–Teller (BET, Quantachrome Autosorb-IQ, Quantachrome, Boynton beach, FL, USA) was used to measure surface properties, including the porosity and the specific surface area of the adsorbent. Thermogravimetric analysis (TGA) of oven-dried powder samples were carried out on a thermogravimetric analyzer (Q500, TA, Newcastle, DE, USA). The hysteresis curve was obtained at room temperature by using a magnetic property measurement system (MPMS SQUID XL, Quantum Design, San Diego, CA, USA). A Shimadzu LC-10AT HPLC system with a RF-20A fluorescence detector (FLD) (Kyoto, Japan) was used for the analysis.

### 3.2. Synthesis of MNCGC

#### 3.2.1. Synthesis of Silica-Coated Fe_3_O_4_

Silica-coated Fe_3_O_4_ was prepared using an alkaline co-precipitation method and sol–gel method. A solution of FeCl_3_•6H_2_O and FeSO_4_•7H_2_O at a molar ratio of 2:1 was dissolved in water and heated to 80 °C under a nitrogen atmosphere with ultrasonic treatment and continuous stirring for 15 min. Ammonia solution was then slowly added dropwise until the pH reached approximately 10. After stirring for an additional 30 min, Fe_3_O_4_ nanoparticles were collected using a magnet and alternately washed three times with water and ethanol until neutral pH was achieved. Finally, the Fe_3_O_4_ nanoparticles were vacuum-dried at 60 °C for 10 h. Next, 0.5 g of Fe_3_O_4_ nanoparticles were added to a mixture of 20 mL water and 100 mL ethanol, followed by continuous stirring and ultrasonication for 30 min to ensure uniform dispersion. Subsequently, 0, 1, 2, 3, and 5 mL of tetraethyl orthosilicate (TEOS) and 4 mL of 25% ammonia solution were slowly added dropwise to the mixture. The reaction was stirred at room temperature (25 °C) for 8 h. The solid was then separated from the liquid using a magnet and washed three times with anhydrous ethanol, followed by vacuum drying at 50 °C for 12 h.

#### 3.2.2. Synthesis of MMC

A mesoporous composite material was prepared using the sol–gel method and etching technique. First, 0.3 g of silica-coated Fe_3_O_4_ was added to a mixture of ethylene glycol and water (1:2, *v*/*v*), vigorously stirred, and ultrasonicated for 10 min to obtain a silica-coated Fe_3_O_4_ suspension. At 60 °C, a certain amount of CTAB was dissolved in the ethylene glycol and water mixture with continuous stirring until fully dissolved. Then, 1 mL of ammonia solution was slowly added dropwise to the silica-coated Fe_3_O_4_ suspension. After stirring at 30 °C for 30 min, 0.1, 0.3, 0.5, 0.7, and 1 mL of TEOS were successively added, followed by 6 h of stirring. The solid and liquid phases were separated using a magnet, and the resulting solid was washed with water to remove any unreacted materials. The solid was then dispersed in 50 mL of water and ultrasonicated for 10 min. Afterward, anhydrous Na_2_CO_3_ was added to adjust the pH to between 10 and 11, and the mixture was stirred at 50 °C for 12 h to complete the etching reaction. The product was collected using a magnet, washed repeatedly with water until neutral, and then vacuum-dried at 50 °C for 12 h to obtain the final product, MMC.

### 3.3. MSPE Procedure

**Figure 11:**
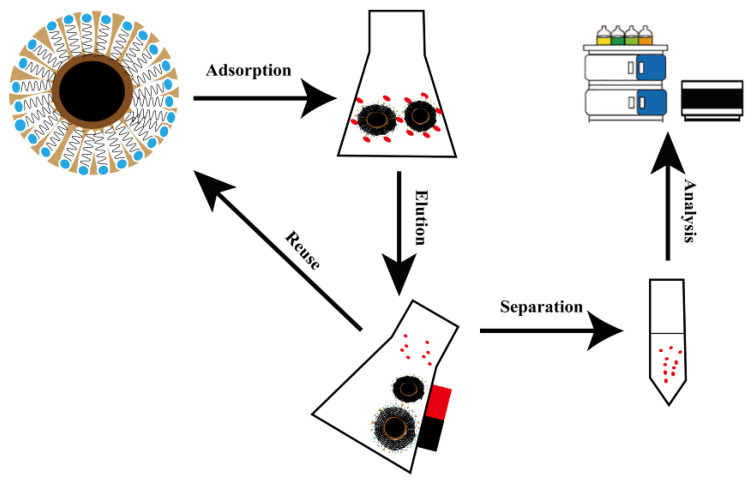
MSPE procedure of MMC.

The process flow diagram for MSPE is shown in Figure 11. A certain amount of MMC was added to a 30 mL solution containing 10 μg/L of six bisphenol pollutants. After uniform dispersion, the mixture was placed in a thermostatic water bath shaker and shaken at 200 rpm at 30 °C for 60 min to complete the extraction. After extraction, the solid and liquid phases were separated using a magnet, and the supernatant was discarded. Then, 5 mL of eluent was added, dispersed evenly, and shaken again at 200 rpm at 30 °C for 60 min in the water bath shaker for elution. After elution, MMC was separated using a magnet, and the supernatant was evaporated to dryness under a nitrogen stream at room temperature. The residue was dissolved in methanol and diluted to a final volume of 500 μL, followed by vortex mixing for 30 s. The solution was then filtered through a 0.22 μm nylon filter and analyzed by HPLC-FLD.

### 3.4. HPLC Analysis

BPA, BPB, BPC, BPF, BPAF, and BPAP were separated and analyzed by a Shimadzu LC-10AT HPLC system (Shimadzu corporation, Kyoto, Japan) with an Agilent C_18_ column (250 × 4.6 mm, 5 μm) and a Shimadzu RF-20A FLD. Mobile phase A was ultrapure water (containing 0.1% formic acid, *v*/*v*); mobile phase B was 100% methanol (containing 0.1% formic acid, *v*/*v*). Gradient elution was used, and the elution program was as follows: 0–10 min, 70% B; 10–20 min, 70–60% B; 20–25 min, 60% B; and 25–30 min, 60–70% B. The flow rate was 0.8 mL/min. The excitation wavelength and emission wavelength were, respectively, set as 227 nm and 313 nm, and the column was controlled at 30 °C. The injection volume was 20 μL.

## 4. Conclusions

In this study, Fe_3_O_4_ was used as the magnetic core, combined with the characteristics of mesoporous materials. CTAB and TEOS were employed as functional monomers, and a simple etching method was used to develop a novel magnetic mesoporous composite material (MMC). The results from SEM, TEM, FTIR, XRD, mapping, EDS, BET, and VSM confirmed that MMC has a wormhole-like mesoporous structure, with an average specific surface area of 148.6 m^2^/g and an average pore size of 2.9 nm. The crystalline structure of MMC was found to be stable, with no signs of aggregation or clumping. The CTAB functional groups were uniformly distributed, and the material exhibited a high level of magnetization, allowing for rapid separation and collection under an external magnetic field during adsorption and desorption processes. Using MMC as the adsorbent in the MSPE process, combined with HPLC-FLD for detection, an analytical method was developed to simultaneously determine trace amounts of six bisphenol pollutants in environmental and food samples. After systematically optimizing the extraction and elution parameters, the final pretreatment conditions were established as a sample volume of 40 mL, 40 mg of CTAB added to 100 mg of MMC adsorbent, pH 7, 1 g/L NaCl, and an extraction rate of 100 rpm for 30 min at 25 °C, followed by desorption with 5 mL of acetone for 10 min at 35 °C and 100 rpm. Through the method was evaluated by testing for interference from humic acid, glucose, fructose, and sucrose, and the results showed minimal interference. The method exhibited good linearity (R > 0.997) over a linear range of 0.05–100 μg/L, with low detection limits (0.01–0.02 μg/L) and quantification limits (0.03–0.05 μg/L). Blank and spiked analyses were performed on seven real samples, including two environmental samples (river and lake water) and three food samples (milk, juice, and carbonated beverages). The spiked recovery rates were satisfactory, ranging from 68.3% to 109.8%. Therefore, the results demonstrate that the established analytical method can successfully detect and analyze trace residues of multiple bisphenol pollutants in various complex matrices, including environmental water and food samples.

## Figures and Tables

**Table 1:** Methodological evaluation of MSPE (MMC)-HPLC-FLD method for the determination of the pollutant of six bisphenols.

BPs	Linear Equation	*R*	LOD	LOQ	Intra-Day RSD (%)	Inter-Day RSD (%)
BPA	0.05–100	0.9998	0.01	0.03	4.19	6.35
BPB	0.05–100	0.9999	0.01	0.03	3.94	5.21
BPC	0.05–100	0.9991	0.01	0.04	4.03	9.29
BPF	0.05–100	0.9976	0.02	0.05	5.14	6.37
BPAF	0.05–100	0.9984	0.01	0.04	4.01	8.43
BPAP	0.05–100	0.9991	0.01	0.05	3.93	7.18

**Table 2:** Comparison of the proposed method with other reported methods.

Method	Adsorbent	Analytes	LODs	Recovery (%)	References
MSPE-HPLC-MS ^a^	Fe_3_O_4_@COF	Bisphenols	0.001–0.078 μg/L	64.8–92.8	[37]
MSPE-HPLC-UV ^b^	Fe_3_O_4_@SiO_2_@Mg-Al-LDH	Bisphenols	0.37–0.63 μg/L	84–103	[38]
MNER-EM ^c^/HPLC-FLD	NiFe_2_O_4_@COF	Bisphenols	0.019–0.096 μg/L	83.4–106.2	[39]
MSPE-HPLC-MS	3DG/ZnFe_2_O_4_	Bisphenols	50–180 ng/L	95.1–103.8	[40]
MSPE-HPLC-MS	MagG@PDA@Zr-MOF	Bisphenols	10–1000 ng/L	64.8–92.8	[41]
MSPE-HPLC-FLD	MMC	Bisphenols	0.01–0.02 μg/L	68.3–109.8	This work

^a^ Mass spectrometry. ^b^ Ultraviolet detection. ^c^ Effervescent reaction-enhanced microextraction.

## Data Availability

Data will be made available on request.

## Supplementary Materials

The following supporting information can be downloaded at: https://www.mdpi.com/article/10.3390/molecules30030628/s1. Figure S1: The adsorption isotherms of BPA on MMC; Figure S2: The adsorption kinetics of BPA on MMC; Figure S3: SEM images of Fe_3_O_4_ (a), silica-coated Fe_3_O_4_ (b), and MMC (c); Figure S4: TEM images of Fe_3_O_4_ (a), silica-coated Fe_3_O_4_ (b), and MMC (c); Figure S5: Mapping image of MMC (a); EDS image of MMC (b); Figure S6: Chromatograms of environmental samples (a) and food samples (b); Table S1: The isotherm parameters of MMC for BPA adsorption; Table S2: MMC adsorption kinetic parameters of BPA; Table S3: Sampling coordinates of environmental water samples; Table S4: Environmental and food sample analysis results.
