# Supplementary material for: Novel Mesoporous Cetyltrimethylammonium Bromide-Modified Magnetic Nanomaterials for Trace Extraction and Analysis of Bisphenol Endocrine Disruptors in Diverse Liquid Matrices
Source: Molecules. 2025 Jan 31;30(3):628. doi: 10.3390/molecules30030628 (PMC11820609; doi:10.3390/molecules30030628)
Supplement: Supplementary file 1 [file molecules-30-00628-s001.zip › molecules-3427618-supplementary.pdf]

2.1. Preparation and Characterization (adsorption types)

To better elucidate the adsorption process of MMC, we performed analyses using six adsorption isotherm models, as shown in the figures S1 and tablesS1. The Langmuir adsorption isotherm model is typically applied to homogeneous adsorption surfaces, indicating that all adsorption sites on the surface have the same adsorption force and that the adsorbed molecules do not interact with one another. On the other hand, the Freundlich adsorption isotherm model assumes that the adsorption sites on the adsorbent surface have different adsorption forces, making the surface heterogeneous. Additionally, the values of RL and n suggest that a value of RL between 0 and 1 indicates that MMC favors the adsorption of BPA, while an n > 1 indicates the preferential adsorption of BPA by MMC.

The Dubinin–Radushkevich (D-R) adsorption isotherm model assumes a non-uniform surface of the adsorbent. Typically, E > 40 kJ/mol indicates a chemical adsorption process, while E < 16 kJ/mol suggests a physical adsorption process. From Tables S1, we can see that E is 947.7 kJ/mol, indicating that the adsorption of BPA by MMC is primarily a chemical adsorption process. Both the Redlich–Peterson and Toth models are three-parameter adsorption isotherms, while the Temkin adsorption isotherm model suggests that the adsorption heat of all adsorbed molecules in the adsorption layer decreases linearly as the surface coverage increases, with the maximum binding energy being evenly distributed during the adsorption process. By comparing the correlation coefficients (R values) of the six isotherm models, the Freundlich model showed a higher R value, suggesting that the Freundlich model may be more suitable for fitting the adsorption isotherm of BPA on MMC, indicating that the adsorption active sites on the MMC surface are heterogeneous. Both Langmuir and D-R models provide theoretical maximum adsorption capacities. Since Langmuir's R value is higher, the maximum BPA adsorption capacity of MMC is calculated to be 208.3 mg/g, which further demonstrates that MMC exhibits a high adsorption capacity for BPA.

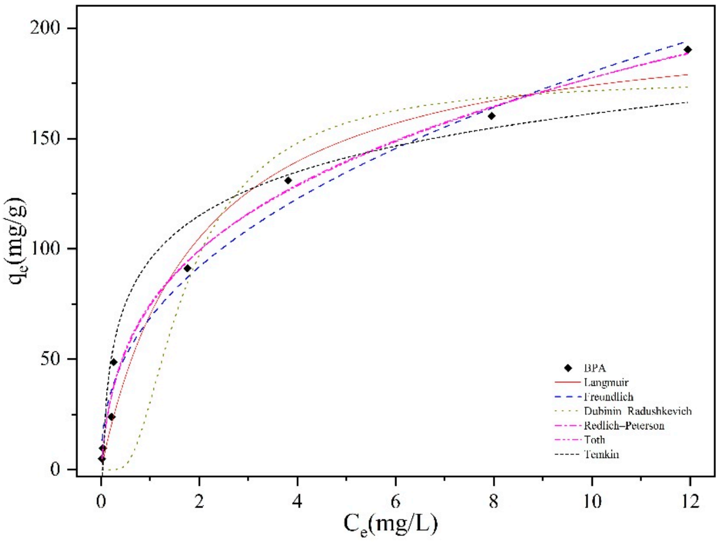

Figure S1. The adsorption isotherms of BPA on MMC.

Table S1. The isotherm parameters of MMC for BPA adsorption.

Model	Parameter	Value
Langmuir	q_{max}（mg/g）	208.3
	K_L（L/mg）	0.51
	R_L	0.03
	R	0.970
Freundlich	n	2.386

Model	Parameter	Value
	K_F（（mg/g）(L/mg)$^{1/n}$）	68.6
	R	0.992
Dubinin–Radushkevich (D-R)	E (KJ/mol)	947.7
	β (mol^2 /J^2)	5.6×10^{-7}
	q_{max}（mg/g）	177.4
	R	0.881
Redlich–Peterson	K_{RP} (L/mg)	358.5
	α_{RP} (L/mg)	3.83
	β_{RP}	0.699
	R	0.989
Toth	K_T (mg/g)	83.28
	a_T (mg/g)	0.172
	t	1.50
	R	0.989
Temkin	B	28.69
	A (L/mg)	27.57
	R	0.923

The adsorption kinetics models reflect the dynamic process of adsorption, helping us to understand the underlying adsorption mechanisms. We employed four different kinetic models to assess the adsorption process of BPA by MMC. The results are shown in the figures S2 and tables S2. The pseudo-second-order kinetic model exhibited a higher correlation coefficient (R), and the calculated adsorption capacity from the model was in good agreement with the actual experimental data. Thus, the pseudo-second-order model provides a good description of the data obtained from the experimental adsorption process.

The initial adsorption rate α of MMC for BPA was 99.84 mg/g·min, indicating that MMC has a considerable BPA adsorption capacity as soon as it is introduced into the solution. The figure S2 shows the linear relationship of intra-particle diffusion during the BPA adsorption process by MMC. As seen from the figure S2, the kinetic data of the adsorption process are not linear, and the adsorption occurs in two distinct stages. From the table S2, it is observed that the K_{int1} parameter value during the first stage is higher than that in the second stage (K_{int2}). The second stage of the adsorption process occurs at a very slow rate, indicating that the rate of diffusion into the interior is limited. Moreover, the I_2 parameter in the second stage is higher than the I_1 parameter in the first stage, suggesting that the thickness of the boundary layer plays a more significant role in the adsorption process during the second stage.

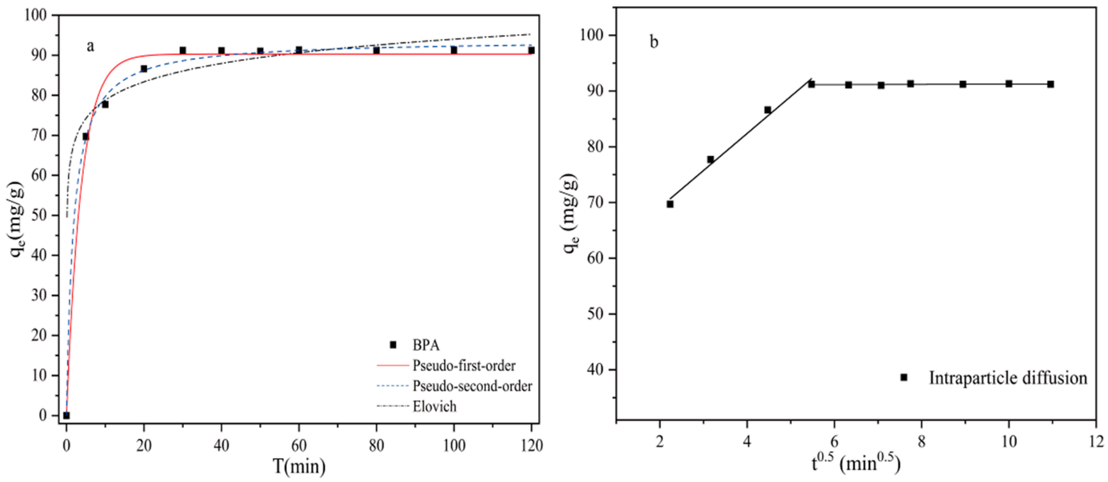

Figure S2. The adsorption kinetics of BPA on MMC.

Table S2. MMC adsorption kinetic parameters of BPA.

Model	Parameter	Value
Pseudo-first-order	k_1（min^{-1}）	0.261
	q_e（mg/g）	90.1
	R	0.996
Pseudo-second-order	k_2(g/mg·min)	0.006
	q_e（mg/g）	93.4
	R	0.999
Elovich	α (mg/g·min)	99.84
	β (g/mg)	0.151
	R	0.986
Intraparticle diffusion	K_{int1}（mg/g $min^{1/2}$）	6.64
	I_1	55.81
	R	0.979
	K_{int2}（mg/g $min^{1/2}$）	0.02
	I_2	91.00
	R	0.989

Based on the adsorption model studies, it was found that the fitting calculation using the D-R adsorption isotherm model yielded an energy value (E) greater than 40 kJ/mol, indicating that the adsorption process of MMC is primarily chemical adsorption. Additionally, the experimental adsorption data fit well with the Freundlich adsorption model, suggesting that MMC has multiple adsorption active sites on its surface, and these active sites are heterogeneously distributed. According to the Elovich and intra-particle diffusion models, it was observed that MMC exhibits a high initial adsorption capacity and rate.

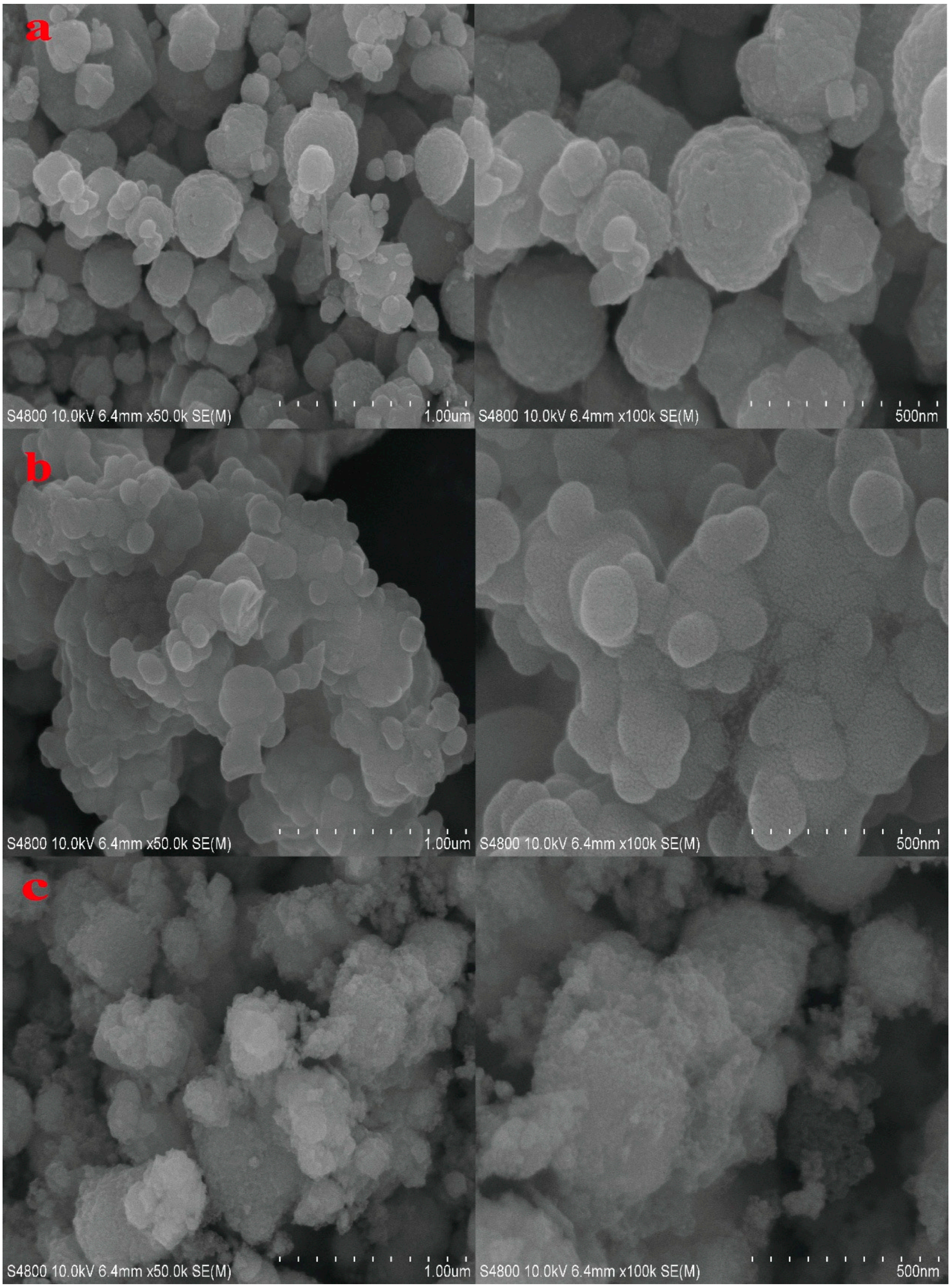

Figure S3. SEM images of Fe_3O_4 (a), silica-coated Fe_3O_4 (b), and MMC (c).

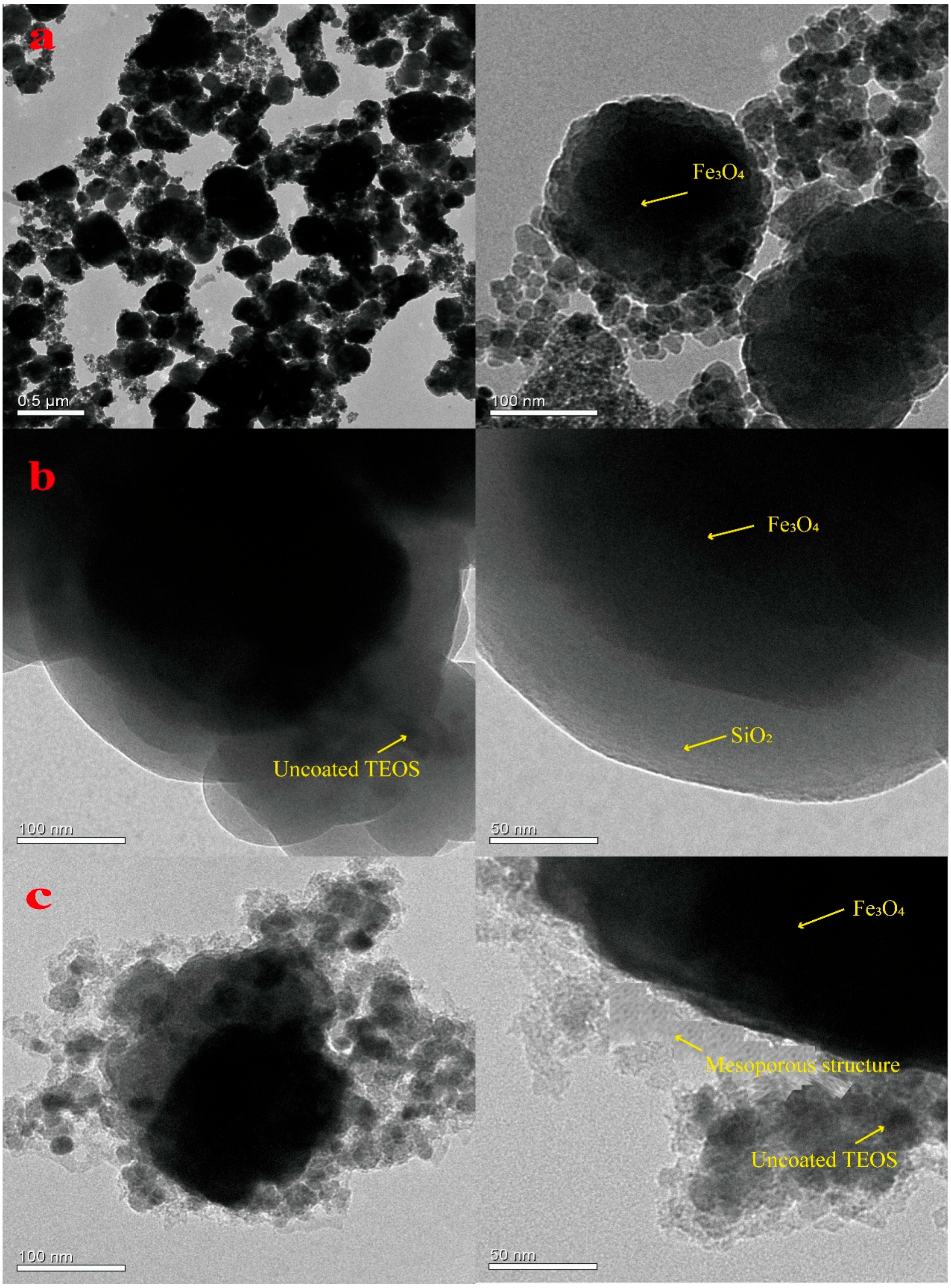

Figure S4. TEM images of Fe_3O_4 (a), silica-coated Fe_3O_4 (b), and MMC (c).

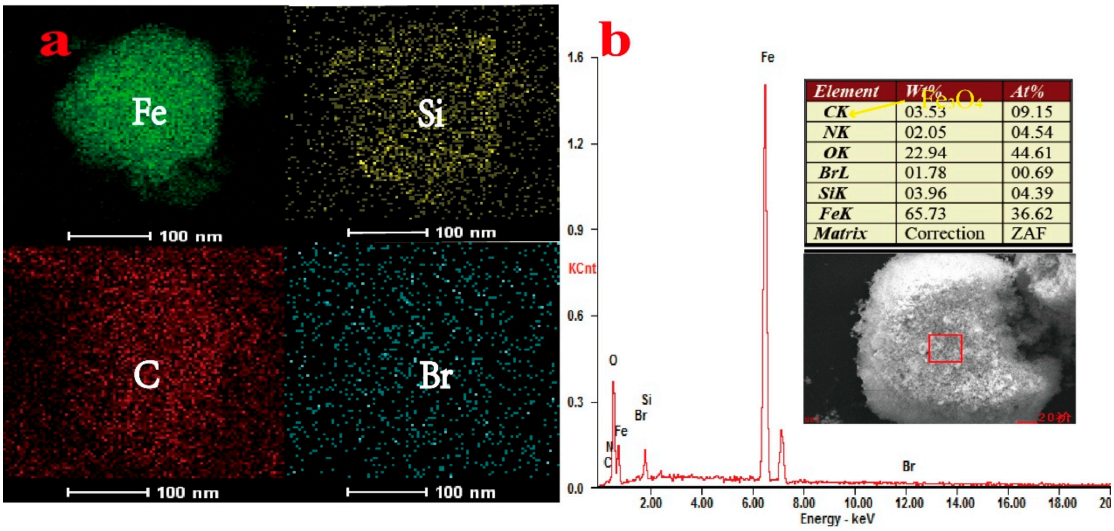

Figure S5. Mapping image of MMC (a); EDS image of MMC (b).

Table S3. Sampling coordinates of environmental water samples.

	Sampling points	Coordinate
Baiyang Lake	S1	115°96'457'' E, 38°84'125'' N
	S2	115°95'141'' E, 38°84'88'' N
Fuhe River	S3	115°30'59'' E, 38°52'10'' N
	S4	115°34'6'' E, 38°52'1'' N

Table S4. Environmental and food sample analysis results.

	Sampling points/Sample	BPA (μg/L)	Recovery (%)	BPB (μg/L)	Recovery (%)	BPC (μg/L)	Recovery (%)	BPF (μg/L)	Recovery (%)	BPAF (μg/L)	Recovery (%)	BPAP (μg/L)	Recovery (%)
Baiyang	S5-1	ND[a]		ND		ND		ND		ND		ND	
Lake	5 (μg/L)		87.6±4.3		89.8±5.9		80.3±4.1		99.7±5.3		92.4±4.7		93.9±3.8
	10 (μg/L)		108.1±6.7		109.3±3.6		100.8±8.3		99.7±4.9		103.1±6.5		104.4±4.6
	S5-2	ND		ND		ND		ND		ND		ND	
	5 (μg/L)		86.3±5.2		78.9±7.7		80.4±4.7		90.1±5.7		83.4±6.1		83.4±6.2
	10 (μg/L)		107.4±5.8		109.1±5.2		94.1±5.4		99.9±3.8		103.7±4.8		103.1±3.7
Fuhe	S5-3	ND		0.21		ND		0.07		ND		ND	
River	5 (μg/L)		88.2±4.9		78.1±9.1		80.3±3.9		80.3±5.2		73.4±9.6		83.7±3.9
	10 (μg/L)		98.7±3.3		99.3±5.8		100.2±4.4		100.4±4.8		102.1±2.9		103.9±4.5
	S5-4	ND		0.30		ND		ND		ND		0.06	
	5 (μg/L)		77.9±8.7		88.3±6.9		81.0±4.1		68.3±9.8		83.3±4.1		84.1±5.8
	10 (μg/L)		108.0±3.8		108.6±5.3		100.8±3.6		98.5±4.1		103.9±6.3		104.7±3.2
Food	Milk	0.17		ND		ND		1.83		0.08		ND	
Samples	5 (μg/L)		99.1±3.3		94.4±3.7		100.7±5.2		99.8±4.7		103.6±6.9		103.4±4.0

Sampling points/Sample	BPA （μg/L）	Recovery (%)	BPB （μg/L）	Recovery (%)	BPC （μg/L）	Recovery (%)	BPF （μg/L）	Recovery (%)	BPAF （μg/L）	Recovery (%)	BPAP （μg/L）	Recovery (%)
10 (μg/L)		109.8±3.1		108.7±5.4		100.3±3.3		100.2±3.7		103.7±4.7		104.4±5.8
Mango juice	0.14		0.09		ND		1.06		ND		ND	
5 (μg/L)		98.8±3.6		98.2±3.2		90.6±4.4		90.3±3.9		102.9±3.2		102.7±3.9
10 (μg/L)		108.4±4.1		108.5±3.8		100.9±4.7		99.1±3.7		103.1±5.0		103.5±5.4
Lemon-flavored soda	0.08		ND		ND		1.41		ND		ND	
5 (μg/L)		97.3±3.6		97.9±5.3		99.3±3.4		100.5±3.3		93.4±4.8		101.1±4.6
10 (μg/L)		98.3±4.9		101.0±2.1		100.5±2.9		99.6±2.7		103.1±3.4		103.5±3.3

[a] Not detected.

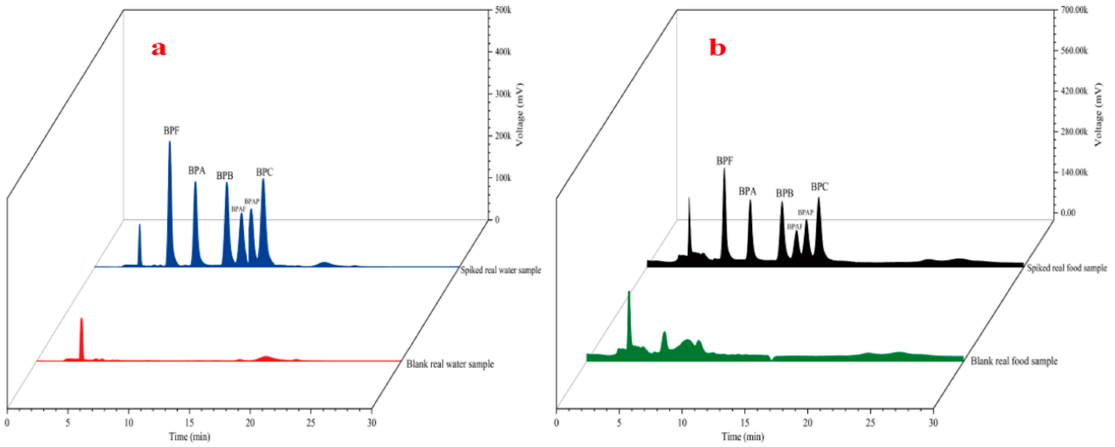

Figure S6. Chromatograms of environmental samples (**a**) and food samples (**b**).
